# Rapid, high-contrast, and steady volumetric imaging with Bessel-beam-based two-photon fluorescence microscopy

**DOI:** 10.1117/1.JBO.29.1.016501

**Published:** 2024-01-24

**Authors:** Yongqiang Chen, Chenggui Luo, Shiqi Wang, Yanping Li, Binglin Shen, Rui Hu, Junle Qu, Liwei Liu

**Affiliations:** Shenzhen University, College of Physics and Optoelectronic Engineering, Key Laboratory of Optoelectronic Devices and Systems of Guangdong Province, Ministry of Education, Shenzhen, China

**Keywords:** nondiffracting beams, two-photon fluorescence microscopy, volumetric imaging, *in vivo* imaging

## Abstract

**Significance:**

Two-photon fluorescence microscopy (TPFM) excited by Gaussian beams requires axial tomographic scanning for three-dimensional (3D) volumetric imaging, which is a time-consuming process, and the slow imaging speed hinders its application for *in vivo* brain imaging. The Bessel focus, characterized by an extended depth of focus and constant resolution, facilitates the projection of a 3D volume onto a two-dimensional image, which significantly enhances the speed of volumetric imaging.

**Aim:**

We aimed to demonstrate the ability of a TPFM with a sidelobe-free Bessel beam to provide a promising tool for research in live biological specimens.

**Approach:**

Comparative *in vivo* imaging was conducted in live mouse brains and transgenic zebrafish to evaluate the performance of TPFM and Bessel-beam-based TPFM. Additionally, an image-difference method utilizing zeroth-order and third-order Bessel beams was introduced to effectively suppress background interference introduced by sidelobes.

**Results:**

In comparison with traditional TPFM, the Bessel-beams-based TPFM demonstrated a 30-fold increase in imaging throughput and speed. Furthermore, the effectiveness of the image-difference method was validated in live biological specimens, resulting in a substantial enhancement of image contrast. Importantly, our TPFM with a sidelobe-free Bessel beam exhibited robustness against axial displacements, a feature of considerable value for *in vivo* experiments.

**Conclusions:**

We achieved rapid, high-contrast, and robust volumetric imaging of the vasculature in live mouse brains and transgenic zebrafish using our TPFM with a sidelobe-free Bessel beam.

## 1 Introduction

**Fig. 1:**
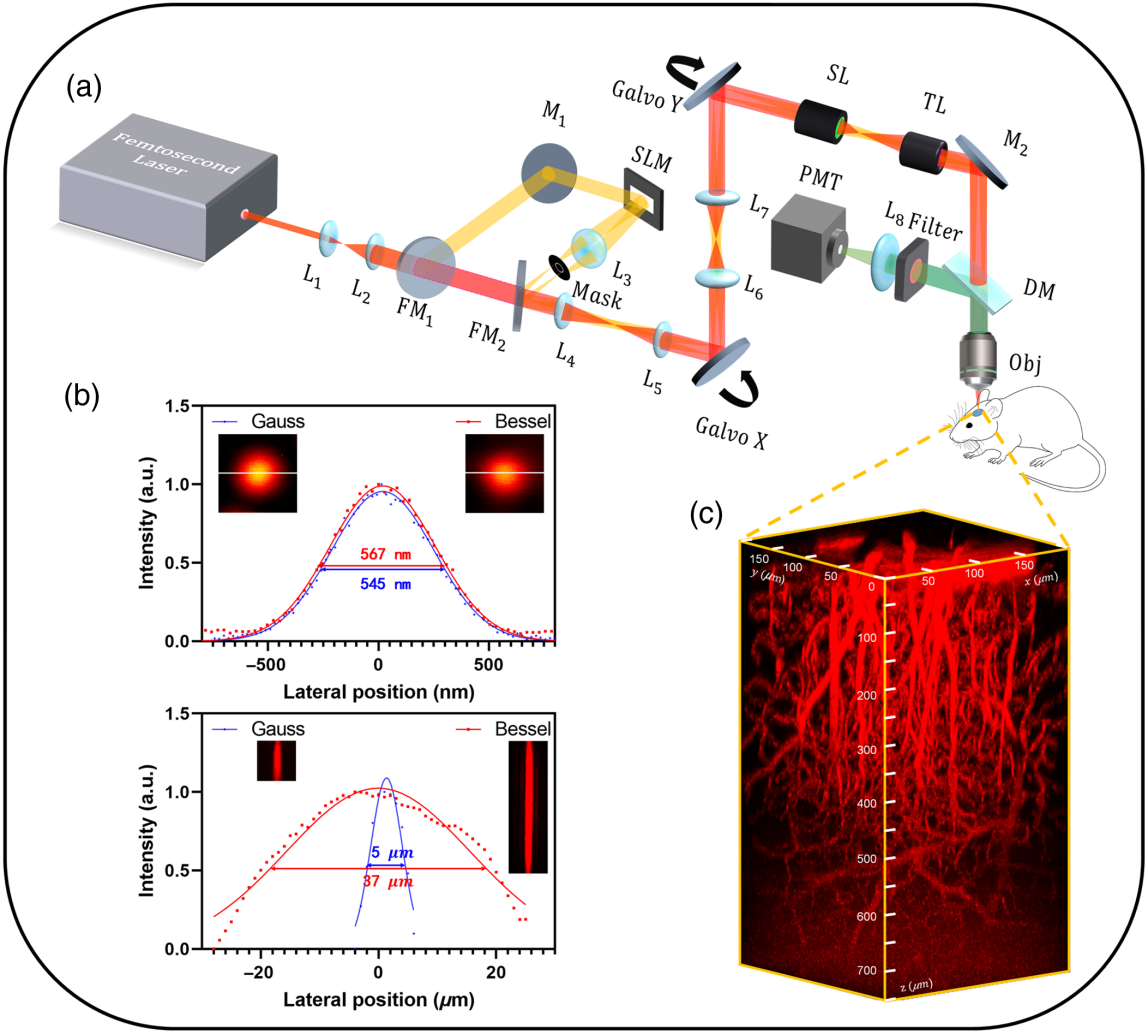
Design of a custom-built two-photon fluorescence microscope with a Bessel/Gaussian beam-switching module. (a) Schematic of the microscope. L, lens; FM, flip mirrors; SLM, spatial light modulation; SL, scan lens; TL, tube lens; Obj, objective; DM, dichroic mirror; and PMT, photomultiplier tube. (b) Lateral and axial resolution for the Gaussian and Bessel focus. Results from a single 0.5-μm-diameter bead. (c) 3D reconstruction of an image stack of mouse brain vasculature scanning by a Gaussian focus.

Most two-photon fluorescence microscopy (TPFM) is typically excited by a Gaussian beam, so it is necessary to perform axial scanning to acquire a three-dimensional (3D) image stack, resulting in a lengthy imaging process. To meet the urgent demand, various efforts have been undertaken in recent decades to develop rapid and high-contrast methods for volumetric imaging,^1^^–^^3^ among which Bessel beams, which have a long nondiffracting distance with a constant lateral resolution,^4^^–^^7^ have shown promise. Bessel-beams-based two-photon fluorescence microscopy (Bessel-TPFM)^8^ has an extended depth of focus (DOF) and greater resilience against scattering, making it well-suited for imaging biological tissues with large axial spans and sparse distributions. For instance, both cerebral blood vessels [Fig. 1(c)] and neurons with extensive dendritic tree structures^9^ may span several hundred microns in depth and remain stationary during imaging. The Bessel-TPFM system can detect a 3D volume at the rate of a two-dimensional (2D) frame, providing abundant imaging throughput and reduced data volume, enabling real-time imaging of single vessels or synapses to local networks, which enables it to span from single vessels or synapses to local networks in real time.

The central lobe of a Bessel beam is surrounded by a series of concentric rings known as sidelobes, which provide energy to maintain the beam’s nondiffracting and self-reconstructing properties. However, the significant power fraction of the sidelobes also introduces strong background noise, reducing image contrast in the imaging process. Therefore, it is essential to suppress the sidelobes while preserving the unique properties of the Bessel beam. Several methods have been proposed to address this challenge, including retaining the sidelobes during excitation while extracting only the signal from the line where the center spot is located during detection, referencing the confocal line principle.^10^ Physical slits^11^^,^^12^ or scientific complementary metal-oxide-semiconductor (CMOS) shutters^13^ can be used to filter out background noise introduced by sidelobes. Higher-order nonlinear effects (such as three-photon excitation),^14^^,^^15^ structured light illumination,^16^ deconvolution algorithms,^17^ and a “Bessel droplet”^18^^,^^19^ with periodic axial light and dark intensity distributions formed by interference of multiple coaxial Bessel beams can also effectively reduce sidelobe effects. Although several methods have been reported to suppress the sidelobes and enhance image contrast, they often involve complex optical setups or image postprocessing. The image-difference method offers a simpler approach by generating two beams to excite the sample and acquiring the difference results of two individual images. One beam is a zeroth-order Bessel beam, and the transverse intensity profile of the other beam should match it to suppress sidelobes via the image-difference method. Jia et al.^20^ used a Mathieu beam as an initial solution and obtained the complementary beam of the zeroth-order Bessel beam through an iterative optimization algorithm. The transverse intensity profile of the third-order Bessel beam meets the condition for the complementary beam of the zeroth-order Bessel beam, allowing for suppression of sidelobe-induced background via the image-difference method.^21^

In most *in vivo* experiments, biological specimens are mechanically fixed to limit sample movement; however, this often still leaves residual motion.^22^ Axial movement will lead to fluctuations and even loss of recorded fluorescence intensity due to changes in the excitation focal plane. Several approaches have been proposed to tackle this challenge, including the use of sedatives^23^ and simultaneous multiplane detection.^24^ The extended DOF of the Bessel-TPFM system also shows promise.

In this paper, we construct a two-photon fluorescence microscope capable of switching between Gaussian and Bessel beam excitation. By comparing an image projection acquired through axial tomographic scanning with a Gaussian focus with a single image frame acquired by scanning with a Bessel focus in a single plane, we demonstrate that the Bessel-TPFM system enables rapid volumetric imaging of *in vivo* vasculature. Moreover, we obtain images of zeroth-order and third-order Bessel beams that have perfectly matched transverse intensity profiles. The resulting difference images effectively reduce the background noise caused by sidelobes. Furthermore, we demonstrate experimentally that the extended DOF of a Bessel beam efficiently counteracts the vertical movements in live biological specimens.

## 2 Materials and Methods

### 2.1 Two-Photon Fluorescence Microscope with a Bessel/Gaussian Module

A schematic diagram of the optical setup is provided in Fig. 1(a). A femtosecond laser (Axon920TPC, Coherent) delivered pulses of up to 1 W and 150-fs pulses at 920 nm with a repetition rate of 80 MHz. After expansion and collimation by an optical 4f system, the laser pulse was directed to a beam-switching device that alternated between the Gaussian and Bessel modules using flip mirrors (FM1 and FM2). In the Bessel module, various orders and DOFs of Bessel beams were generated by a phase-only spatial light modulator (SLM, Pluto NIR-015, Holoeye) and then transformed into optical rings of diverse radii by L3. A custom annular mask^25^ was placed at the back focal plane of L3 to filter out stray light outside the Bessel beam. The optical rings were relayed to a pair of galvanometer scanning mirrors (GVS011/M, Thorlabs) through optical 4f systems (L4 to L7), with the scanning mirrors conjugated with the rear pupil of the microscope objective using a scan lens and a tube lens. Objective1 (Plan Apo Lambda 20×, Nikon) was used to measure fluorescent beads, and objective2 (XLPLN25XWMP2, 25×, NA 1.05, Olympus) was used for *in vivo* experiments. The fluorescence emission from the sample was collected by the same objective and passed through a dichroic mirror (ZT670rdc-xxrxt, Chroma), a short-pass filter (ET680sp-2p8, Chroma), and a collection lens to finally reach the photomultiplier tube (PMT2001/M, Thorlabs).

### 2.2 Sample Preparation

All animal operations complied with the protocols approved by the Institutional Ethical Committee of Animal Experimentation of Shenzhen University. C57 mice were purchased from Guangdong Medical Laboratory Animal Center. Before 2-photon imaging, craniotomies were performed on C57 mice (female, 8 to 10 weeks old, weighing 20 to 21 g) centered at 2 mm lateral and posterior to the bregma point. A cover glass was used to seal the cranial window. To fix the mice, a home-made alloy ring was stuck onto the skull by dental cement. During surgery and imaging, a gaseous anesthesia system (Matrix VIP 3000, Midmark) and a heating blanket were used to anesthetize mice and maintain their body temperature, respectively. A prepared aqueous solution (200  μL) of fluorescent probes TPETPABT^26^ was injected through the orbit to label the brain vasculature.

Zebrafish embryos of the Tg(kdrl:EGFP) strain were obtained from the China Zebrafish Resource Center and raised in E3 solution containing 0.003% N-phenylthiourea (Sigma) to inhibit pigmentation after 20 h postfertilization. Vascular epithelial cells were labeled with EGFP. Prior to live imaging, zebrafish were anesthetized with 600  μM Tricaine (Sigma, Cat. # E10521) and mounted in 1% low-melting-point agarose (NuSieve GTG, Cambrex BioScience, Cat. # 50080) for imaging to minimize movement artifacts during imaging.

### 2.3 SLM-Based Bessel Module

Herein, we used a phase-only SLM^27^ instead of an axicon to generate Bessel beams. The SLM can produce tunable Bessel beams, allowing for modulation of the order, lateral resolution, and depth of focus to adapt to different samples and applications. To generate Bessel beams using an SLM, a grayscale image must be loaded onto the device. This image can be a hologram encoded with either an axicon or an annular aperture, with the former having a higher beam conversion efficiency:^28^

The transmission function of the axicon^29^ is given as

t(ρ,φ)=exp[ikα(n−1)ρ]exp(ilφ), (1)

where (ρ,φ) is the polar coordinate; k is the wavenumber; n and α are the refractive index and cone angle of the axicon, respectively; and l is the topological charge, which indicates its order. Thus the mathematical expression for the axicon-encoded hologram is

ϕ=mod[kα(n−1)ρ+lφ,2π]. (2)

In Fig. 1, the Fourier spectral plane of the Bessel beam (i.e., the mask) is conjugated to the pupil plane of the objective. The effective numerical aperture ${\mathrm{NA}}_{\mathrm{eff}}$ of the Bessel focus scanned on the sample depends on the annular radius $R_{\text{pupil}}$ on the pupil plane of the objective:

$${\mathrm{NA}}_{\mathrm{eff}}=\frac{R_{\text{pupil}}}{f_{\mathrm{obj}}},$$ (3)

$$R_{\text{pupil}}=mf_{3}\;\sin\;\beta .$$ (4)

Here $f_{\mathrm{obj}}$ is the effective focal length of the objective, m is the magnification from the mask to the pupil of the objective, $f_{3}$ is the focal length of the lens after the SLM, and $\beta =\sin^{-1}(n\;\sin\;\alpha )-\alpha$.

The lateral resolution of the generated Bessel focus is given as

$$\rho =\frac{2.4048\lambda f_{\mathrm{obj}}}{2\pi R_{\text{pupil}}}=\frac{2.4048\lambda }{2\pi {\mathrm{NA}}_{\mathrm{eff}}},$$ (5)

and its DOF is

$$L=\frac{r_{\mathrm{obj}}f_{\mathrm{obj}}}{R_{\text{pupil}}}=\frac{r_{\mathrm{obj}}}{{\mathrm{NA}}_{\mathrm{eff}}},$$ (6)

where λ is the wavelength of the incident light and $r_{\mathrm{obj}}$ is the diameter of the pupil of the objective.

Both lateral resolution and the DOF are inversely proportional to the ${\mathrm{NA}}_{\mathrm{eff}}$, i.e., they counterbalance each other. The DOF of the Bessel focus can be modulated by the hologram loaded on SLM, the lens positioned after the SLM, the magnification from the mask to the objective’s pupil, and the objective itself. Although the enhancement can exceed 30-fold, it would necessitate a substantial increase in power usage. Moreover, an overly aggressive enhancement could potentially lead to signal overlap. Thus we produced a Bessel focus with a lateral resolution slightly lower than that of the Gaussian focus but with a DOF approximately seven times greater [Fig. 1(b)], wherein α=1  deg, $f_{3}=75\;\mathrm{mm}$, m=6, and objective 1 were used.

## 3 Results

### 3.1 Rapid Volumetric Imaging with the Bessel-TPFM System

**Fig. 2:**
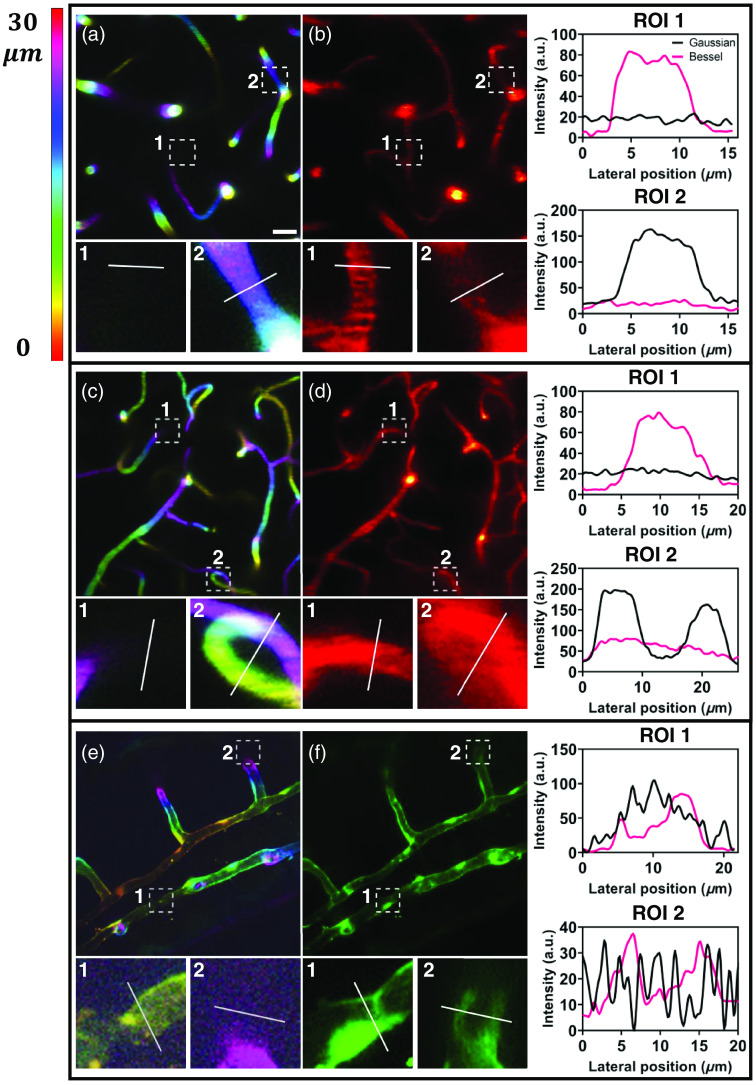
*In vivo* volumetric structural imaging of vasculature using the Bessel-TPFM system. Gaussian-TPFM image projection of vasculature at a depth of (a) 300 to 330  μm and (c) 400 to 430  μm below the top surface of the dura mater in the mouse cortex *in vivo*, color-coded by depth. (e) Gaussian TPFM image projection of vasculature in the Tg (kdr: EGPF) s843 transgenic zebrafish, expressing GFP in vascular endothelial cells, color-coded by depth. (b), (d), and (f) Scanning the Bessel focus in 2D captured all vasculature in the volumes shown in (a), (c), and (e), respectively. The regions of interest (ROIs) were enlarged from the areas within the dashed boxes in (a)–(f), and the intensity profiles were compared across the same blood vessels. Scale bars: 20  μm. Postobjective power: (a) 15 mW, (b) 45 mW, (c) 40 mW, (d) 80 mW, (e) 10 mW, and (f) 52 mW.

To demonstrate the capabilities of fast volumetric imaging, we used our home-built Bessel-TPFM system for performing volumetric imaging of 200  μm×200  μm areas of vasculature in the mouse brain and transgenic zebrafish using both Gaussian and Bessel foci (Fig. 2). The 3D projection images were obtained by axial scanning with a step size of 1  μm using a Gaussian focus, with depth information color-coded to enable *post hoc* assignment of depth information to data from the Bessel module. Obviously, a 2D image obtained by scanning with a Gaussian focus can only capture the transverse section of a blood vessel, so axial scanning is required to capture the complete blood vessel signal. For *in vivo* volumetric imaging, we scanned a volume of 200  μm×200  μm×30  μm using a Gaussian focus and acquired a stack of 30 images, with each 512×512 frame taking 5 s, which is not sufficient for real-time monitoring in *in vivo* imaging. By contrast, a single-frame obtained by 2D scanning with a Bessel focus can capture all signals within the volume, increasing the imaging throughput by 30 times. The speed enhancement can be quantified as N, which is the ratio of the DOF of the Bessel focus by the axial step of the Gaussian focus. In this study, the DOF of the Bessel focus is 37  μm and the axial step of the Gaussian focus is 1  μm. Considering the problem of weak signal loss, the speed of the Bessel-TPFM system can be augmented by more than 30 times. It should be mentioned that the imaging speed here is limited by the hardware conditions of the galvanometer, whereas the fast volumetric imaging capability of the Bessel-TPFM system is compatible with fast scanning devices, such as resonant scanners and acousto-optic deflectors. In addition, during tomographic scanning, the step size causes some signals to not be detected by the Gaussian projection, such as in the ROI 1 of Figs. 2(a)–2(d), resulting in the loss of some detailed biological information. The postobjective power utilized in Figs. 2(a)–2(f) was 15, 45, 40, 80, 10, and 52 mW, respectively. Bessel foci require significantly more power to generate the same intensity as they distribute the majority of their energy into their sidelobes and have extended DOFs. However, due to the large fraction of power in the sidelobes of the Bessel beam, the signal-to-background ratio (SBR) of the Bessel frame in the same areas is lower than the Gaussian projection that averages dozens of frames, such as ROI 2 of Figs. 2(a)–2(d). From the imaging results of zebrafish [ROIs of Figs. 2(e)–2(f)], the image quality of the Bessel frame is better than that of Gaussian projection. This may be due to the fact that zebrafish embryos, as transparent samples, have less scattering and sufficient power (5 times that of Gaussian projection) to excite a strong enough signal, making the background produced by sidelobes less noticeable. However, the vast majority of biological samples are strongly scattering and opaque substances, and high power is limited in biological microscopy. Therefore, it is necessary to further suppress side lobes that introduce background noise.

### 3.2 High-Contrast Imaging with the Bessel-TPFM System

**Fig. 3:**
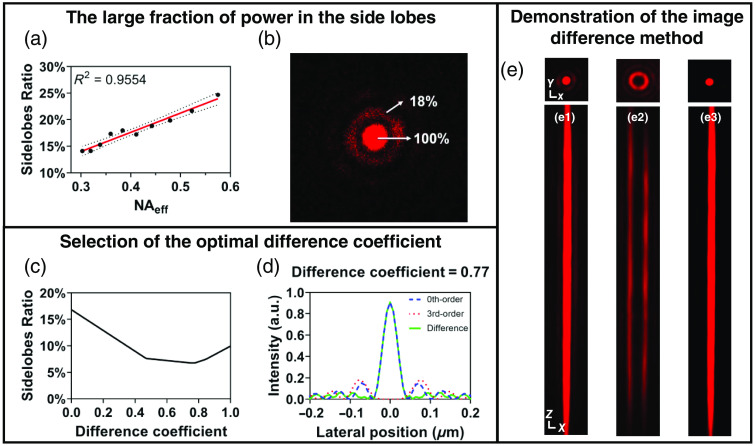
Proportion of energy in the side lobes and the image-difference method. (a) The proportion of energy in the first side lobe relative to the central main lobe under different ${\mathrm{NA}}_{\mathrm{eff}}$. Error dotted lines represent standard deviations. Experimental data were linearly fitted. (b) The proportion of energy in the x−y cross section of the Bessel beam when ${\mathrm{NA}}_{\mathrm{eff}}=0.38$. (c) The proportion of energy in the side lobes after image difference processing with different difference coefficients. (d) The simulated results of the image-difference method. (e) The lateral and axial intensity distributions of the (e1) zeroth order, (e2) third-order Bessel beam, and (e3) the difference result. X, Y, and Z scale bars: 1  μm. Results from one 1-μm-diameter bead.

We imaged fluorescent beads using the constructed Bessel-TPFM system and acquired the sidelobe proportion of Bessel beams with different effective numerical apertures ${\mathrm{NA}}_{\mathrm{eff}}$ [Fig. 3(a)]. The sidelobe proportion is represented by the energy ratio of the first-order sidelobe to the central main lobe. Although two-photon excitation can suppress the energy of sidelobes to a certain extent compared with single-photon excitation, it can be seen that, even in the case of low NA, the sidelobe proportion is still close to 20% [Fig. 3(b) shows the imaging result when ${\mathrm{NA}}_{\mathrm{eff}}=0.38$] and it rises with the increase of ${\mathrm{NA}}_{\mathrm{eff}}$. For high-resolution imaging, the situation of high ${\mathrm{NA}}_{\mathrm{eff}}$ must be considered.

Because the intensity distribution of the third-order Bessel beam is roughly the same as the sidelobe distribution of the zeroth-order Bessel beam, by selecting an appropriate coefficient to perform a difference between the two, the sidelobe energy can be greatly reduced while maintaining the central spot. The difference outcome is derived from the subtraction of the third-order Bessel beam image from the zeroth-order Bessel beam image. Given the disparity in their amplitudes, it is imperative to incorporate a differential coefficient to bridge this gap. We identified the optimal difference coefficient that results in the minimization of the energy proportion of the first sidelobe. As shown in Fig. 3(c), we compared the suppression effect of sidelobes under different difference coefficients through numerical simulation. The sidelobe proportion is the energy ratio of the first-order sidelobe to the central spot after taking the absolute value of the difference results. When the difference coefficient is 0.77, the sidelobe proportion decreases from 16.8% to 6.77% [Fig. 3(d)].

We generated zeroth-order and third-order Bessel foci of ${\mathrm{NA}}_{\mathrm{eff}}=0.38$ to scan the fluorescent beads and calculated their difference result with the optimal difference coefficient [Fig. 3(e)]. It is obvious that the sidelobe distribution of the zeroth-order Bessel beam matches well with the transverse intensity distribution of the third-order Bessel beam and the axial length is also basically consistent. Therefore, the signals from the central spot are maintained whereas those from the sidelobes are effectively suppressed after image difference processing.

Although previous research^21^ has introduced the image-difference method based on Bessel beams with successful applications in mouse brain sections, tissue slices lack rapidly changing physiological information, eliminating the need for sacrificing depth information for rapid volume imaging using the Bessel focus. Bessel-TPFM represents a technique that enhances imaging throughput at the expense of depth information and is aimed at studying rapidly changing physiological activities *in vivo*. To validate the effectiveness of this method in more varied and complex physiological environments, we extended its application to the *in vivo* imaging of mouse brains and zebrafish.

**Fig. 4:**
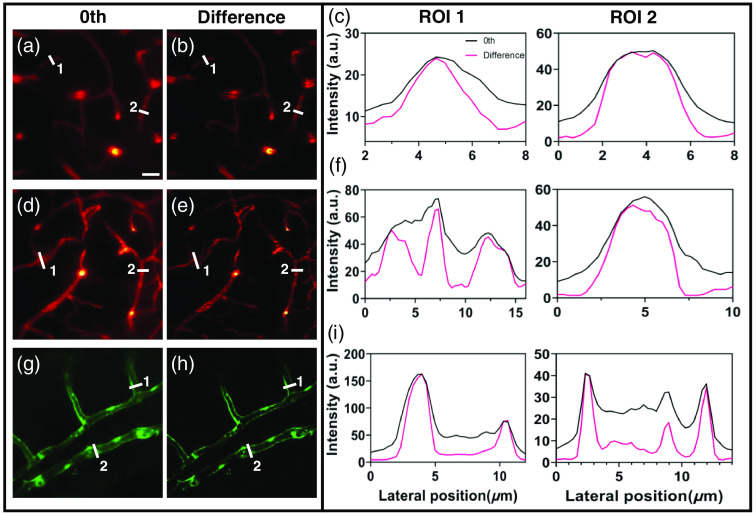
Image-difference method in the Bessel TPFM system. (a), (d), and (g) Image of the vasculature in mouse brain (red) and zebrafish (green) scanned by zeroth-order Bessel beam. (b), (e), and (h) The difference results. (c), (f), and (i) The intensity profiles of ROIs in (a), (b), (d), (e), and (g), (h). Scale bars: 20  μm.

To suppress the background in the Bessel frames [Figs. 4(a), 4(d), and 4(g)], we used the image-difference method with a difference coefficient of 0.5, which is distinct from the optimal theoretical value. However, the optimal theoretical value is computed with the objective of minimizing the first sidelobes, while not taking into account the potential degradation of peak signal and the occurrence of image artifacts. The difference results exhibit clearer and more distinct vessel contours without significant signal loss [Figs. 4(b), 4(e), and 4(h)]. To further elucidate the changes in image contrast via the image-difference method, we selected ROIs at different vessels and measured the intensity profiles by drawing lines. In the difference results [ROIs of Figs. 4(b), 4(e), and 4(h)], the signal peaks of the vessels become significantly narrower, whereas the background signal is efficiently suppressed, allowing for improved separation between signal peaks. The SBR is markedly enhanced, demonstrating that the image-difference method can effectively suppress background noise generated by the side lobes of Bessel beams, thereby improving imaging contrast. Notably, we selected the same regions in both Figs. 2 and 4, enabling a comparison of the results from the Gaussian and Bessel modules, as well as the image-difference method.

### 3.3 Consistently Steady Imaging With the Bessel-TPFM System

Axial movement caused by heartbeats and breathing presents a significant challenge in *in vivo* measurements. The Bessel-TPFM system exhibits robustness against axial motion artifacts due to its extended DOF,^30^ enabling it to steadily monitor changes in fluorescence signals during real-time *in vivo* measurements. We initially observed Bessel beams’ stability in living samples, which is a promising approach for biomedical research.

**Fig. 5:**
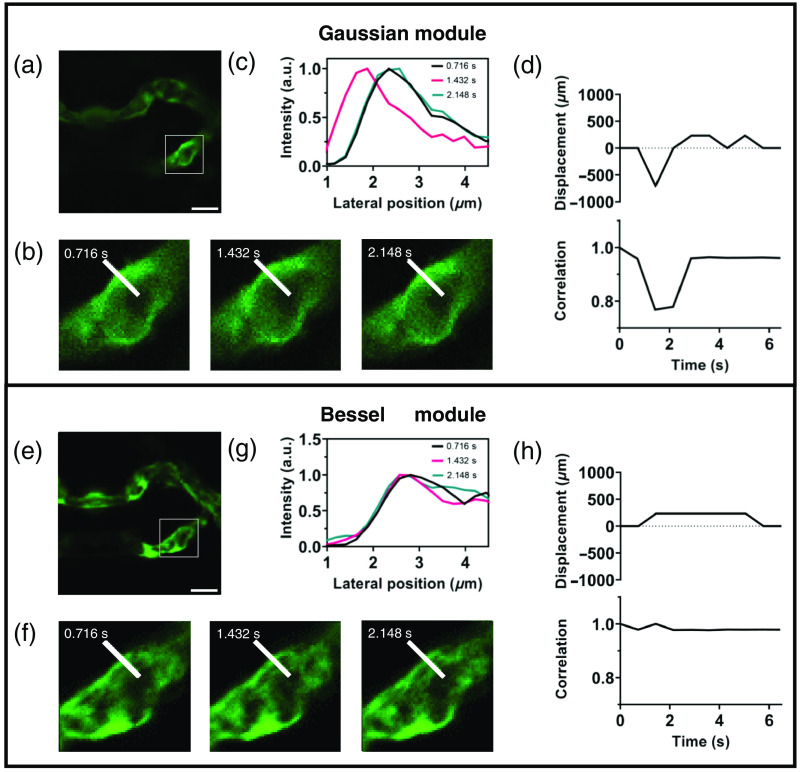
Image time series of the vasculature in zebrafish scanned by (a) Gaussian foci and (e) Bessel foci. (b) The ROIs extracted from (a). (c) The intensity profiles along the white line in (b). (d) The displacement and correlation of the ROIs in the image time series. (f) The ROIs extracted from (e). (g) The intensity profiles along the white line in (f). (h) The displacement and correlation of the ROIs in the image time series. Scale bars: 10  μm (Video 1, MP4, 38.8 kB [URL: https://doi.org/10.1117/1.JBO.29.1.016501.s1]; Video 2, MP4, 43.4 kB [URL: https://doi.org/10.1117/1.JBO.29.1.016501.s2]).

In Fig. 5, we used the Gaussian module and Bessel module to acquire image time series of the same region of zebrafish blood vessels [Fig. 5(a) shows a representative frame from Video 1, and Fig. 5(e) displays a representative frame from Video 2]. For the Gaussian module, the vertical displacement of blood vessels caused signal drift and loss of detail [Figs. 5(b) and 5(c)], resulting in measurement bias. In Fig. 5(d), we calculated the displacement and correlation coefficient between each pair of adjacent images in the image time series, demonstrating that vibration of blood vessels can cause significant measurement bias. By contrast, the Bessel module exhibited a degree of resistance to axial displacements, allowing for better preservation of signal details [Fig. 5(f)] and consistently steady monitoring of fluorescence signals [Figs. 5(g) and 5(h)].

## 4 Discussion and Conclusion

In this study, we aimed to achieve real-time *in vivo* imaging monitoring using a SLM to generate Bessel foci for volumetric imaging at high frame rates while maintaining stable lateral resolution. Our results showed that, when compared with Gaussian projection, the Bessel-TPFM system demonstrated fast volumetric imaging capability under sufficient power and signal-to-noise ratio. Specifically, at 2 to 5 times the power of the Gaussian module, the imaging throughput of the Bessel-TPFM system could be increased by 30 times. Despite the increased power requirement, photodamage and photobleaching caused by Bessel foci were not significantly more severe than those caused by Gaussian foci. This is because Bessel foci have extended DOFs and a wide distribution of energy in the sidelobes. The fluorescence intensity generated by Bessel foci does not show a significant increase, and there are no apparent changes in vascular morphology and fluorescence intensity under long-term observation, which also indicates that the laser power used will not cause additional damage to biological samples. Additionally, we employed an image-difference method using zeroth-order and third-order Bessel beams images to suppress sidelobe-introduced background. Both numerical simulations and experimental results demonstrated that this method performed excellent suppression effects at ${\mathrm{NA}}_{\mathrm{eff}}=0.38$, with even better suppression achievable at higher NAs where sidelobes have a higher-energy contribution. Although the method requires additional image data and postprocessing, it still has significantly higher information throughput and less time compared with the Gaussian-based images. Compared with complex optical system and more complicated image postprocessing methods, this method has a great advantage in its simple operation. Furthermore, we found that the extended DOFs of Bessel foci could avoid dramatic changes due to vertical vibrations or focus drift, providing consistently stable measurements for *in vivo* imaging. In conclusion, our Bessel-TPFM system and image-difference method achieved fast, high-contrast, and steady volumetric imaging in live biological specimens.

## Data Availability

Data and code underlying the results presented in this paper are not publicly available at this time but may be obtained from the authors upon reasonable request.
